# Comparison of Influenza-Like Illness (ILI) incidence data from the novel LeCellPHIA participatory surveillance system with COVID-19 case count data, Lesotho, July 2020 – July 2021

**DOI:** 10.1186/s12879-023-08664-4

**Published:** 2023-10-16

**Authors:** Sarah D. Francis, Gerald Mwima, Molibeli Lethoko, Christiana Chang, Shannon M. Farley, Fred Asiimwe, Qixuan Chen, Christine West, Abigail R. Greenleaf

**Affiliations:** 1 Department of Epidemiology, Mailman School of Public Health, Columbia University, New York, USA; 2 ICAP at Lesotho, Maseru, Lesotho; 3 ICAP at Columbia, New York, USA; 4 Department of Population and Family Health, Mailman School of Public Health, Columbia University, New York, USA; 5 CDC, Maseru, Lesotho; 6 Department of Biostatistics, Mailman School of Public Health, Columbia University, New York, USA; 7 Centers for Disease Control (CDC), Atlanta Global Health Center/Division of Global HIV and TB, Atlanta, USA

**Keywords:** Participatory surveillance, COVID-19, Influenza-like-Illness

## Abstract

**Background:**

While laboratory testing for infectious diseases such as COVID-19 is the surveillance gold standard, it is not always feasible, particularly in settings where resources are scarce. In the small country of Lesotho, located in sub-Saharan Africa, COVID-19 testing has been limited, thus surveillance data available to local authorities are limited. The goal of this study was to compare a participatory influenza-like illness (ILI) surveillance system in Lesotho with COVID-19 case count data, and ultimately to determine whether the participatory surveillance system adequately estimates the case count data.

**Methods:**

A nationally-representative sample was called on their mobile phones weekly to create an estimate of incidence of ILI between July 2020 and July 2021. Case counts from the website Our World in Data (OWID) were used as the gold standard to which our participatory surveillance data were compared. We calculated Spearman’s and Pearson’s correlation coefficients to compare the weekly incidence of ILI reports to COVID-19 case count data.

**Results:**

Over course of the study period, an ILI symptom was reported 1,085 times via participatory surveillance for an average annual cumulative incidence of 45.7 per 100 people (95% Confidence Interval [CI]: 40.7 – 51.4). The cumulative incidence of reports of ILI symptoms was similar among males (46.5, 95% CI: 39.6 – 54.4) and females (45.1, 95% CI: 39.8 – 51.1). There was a slightly higher annual cumulative incidence of ILI among persons living in peri-urban (49.5, 95% CI: 31.7 – 77.3) and urban settings compared to rural areas. The January peak of the participatory surveillance system ILI estimates correlated significantly with the January peak of the COVID-19 case count data (Spearman’s correlation coefficient = 0.49; *P* < 0.001) (Pearson’s correlation coefficient = 0.67; *P* < 0.0001).

**Conclusions:**

The ILI trends captured by the participatory surveillance system in Lesotho mirrored trends of the COVID-19 case count data from Our World in Data. Public health practitioners in geographies that lack the resources to conduct direct surveillance of infectious diseases may be able to use cell phone-based data collection to monitor trends.

**Supplementary Information:**

The online version contains supplementary material available at 10.1186/s12879-023-08664-4.

## Introduction

Since the emergence of the SARS-CoV-2 virus, public health surveillance data have played a key role in tracking and responding to the virus [1]. Surveillance data help determine when and among whom outbreaks are occurring, and identify priority locations for resources and medical personnel allocation [1]. Laboratory testing in a representative sample is the gold standard method for surveilling the incidence of COVID-19 in a population [2]. However, laboratory testing is often limited and requires time to build, particularly in resource limited settings [3].

In Lesotho, a landlocked country of 2.1 million people in Southern Africa, the capacity to conduct laboratory testing for COVID-19 has been limited [4, 5]. According to the COVID-19 online dashboard Our World in Data (OWID), which aggregates COVID-19 case count data based on local media and government reports, Lesotho recorded a cumulative 34,490 COVID-19 cases and 697 deaths between May 13, 2020 and September 22, 2023, experiencing a peak in cases between late December 2020 and late February 2021 and a second peak in June 2021.

Lesotho did not conduct COVID-19 testing consistently at the beginning of the pandemic [5]. Thus, implementing alternative methods to estimate disease incidence in the population was needed [6]. One such option was participatory surveillance; when a population at risk reports on their health via technology that is independent of the health care system [7]. This active participation of community members is a promising method to supplement existing surveillance systems in the context of a pandemic and has been used in other contexts of disease outbreaks to provide real-time, low-cost data on disease spread [3, 8–12].

“LeCellPHIA”, a cell phone-based surveillance system that calls participants weekly to inquire about Influenza-likeIllness (ILI) symptoms (fever, dry cough, and shortness of breath), is an example of a participatory surveillance system. In other settings, surveillance of ILI has been an effective tool for early detection of COVID-19, and is now possible in sub-Saharan Africa given cell phones are increasingly common [13–15]. To establish the ability of LeCellPHIA to capture ILI trends (as a proxy for COVID-19 incidence), we compared LeCellPHIA ILI estimates with the reference standard COVID-19 data, OWID. *This paper aims* to *provide a proof of concept showing that cell phone based participatory surveillance is useful for monitoring infectious disease outbreaks in resource limited settings.*

## Methods

### LeCellPHIA study design and participants

The LeCellPHIA cell phone-based participatory surveillance system was built from the 2020 Lesotho Population-Based HIV Impact Assessment (LePHIA2020) survey [16]. LePHIA2020 was a household-based, cross-sectional nationally representative survey conducted from December 2019 to March 2020 that used two-stage sampling to select respondents. LePHIA2020 assessed the prevalence of key human immunodeficiency virus (HIV) related health indicators. Details on the LePHIA2020 can be found in the Lesotho PHIA final report [17].

To build LeCellPHIA, all 342 primary sampling units from the 10 LePHIA2020 districts were sampled. To ensure we had a sufficient number of older adults in our sample, who are at higher risk of COVID-19 mortality, households with elderly persons (defined as age $\ge$ 60) were oversampled with a ratio of 2:1 between households with and without elderly. From each sampled household, one LePHIA2020 adult participant was randomly sampled from amongst those who consented to future research and provided a valid cell phone number in the LePHIA study. To be eligible, participants had to reside in the same household where LePHIA2020 was conducted. Among those who were eligible and consented, the interviewer confirmed which of their household members listed during LePHIA2020 were still living with the participant. If the randomly selected household member was still present, the participant was called each week and asked to report on their ILI symptoms. This participant was asked throughout the study to provide a proxy report on all household members that participated in LePHIA and that they had seen in the past week. Only participants 15 years and older were included in our study.

LeCellPHIA participants were enrolled using a three-step consent process. The initial consent included information about the purpose of the study, described participant requirements, clarified that participation is voluntary and outlined the anticipated length of the study. The second consent was verbal and consented participants to weekly calls to ask about ILI symptoms. If the participant consented to this consent, the third consent asked if the participant agreed for the interviewer to contact a household member in the event that the interviewer was unable to reach the participant.

Among those sampled, 68% enrolled (AAPOR Response Rate #2) [18]. Beginning July 15, 2020, interviewers called participants weekly and asked whether they or any member of their household had experienced Influenza-Like Illness (ILI) symptoms (fever, dry cough, shortness of breath) over the past week. The response rate for this 12-month period was 75% and weekly response rates ranged from 68 to 88% [19].

### Our World in Data

COVID-19 data was not available from the Ministry of Health in Lesotho. We sought other sources of comparison data and after considering various sources, we determined Our World in Data (OWID) as the best source. The OWID website launched in 2014 and is updated daily, publishing international data on health outcomes, including daily new confirmed COVID-19 cases [20]. The OWID COVID-19 dataset is a publicly available collection of COVID-19 data published by the COVID-19 Data Repository by the Center for Systems Science and Engineering at Johns Hopkins University. Sources for the website include Twitter feeds, online news services, and direct communication sent to the dashboard; case numbers are confirmed with regional and local health departments [21]. We extracted Lesotho’s daily COVID-19 case count data for this analysis.

### Measures

The LeCellPHIA system captures the incidence of ILI each week. To calculate the weekly LeCellPHIA ILI incidence rate, any report of ILI symptoms (fever, dry cough, or shortness of breath) was included in the numerator. The denominator was comprised of participants (including reports from participants about household member symptoms) who answered the symptom questions that week. All results are weighted to adjust for unequal probability of selection, non-response, and potential under-coverage of sampling frame. SAS (version 9.4; SAS Institute) and R Studio (version 2022.07.0; RStudio, PBC) were used to conduct all analyses. Gender, age, and location data were obtained from LePHIA2020.

To calculate the weekly number of confirmed COVID-19 reports recorded in the OWID surveillance system, daily OWID reports of COVID-19 were aggregated into seven-day periods, corresponding with the weekly dates used by the LeCellPHIA surveillance system. LeCellPHIA dates were then roughly matched to Epi Weeks. The surveillance system collected data for all weeks between July 22, 2020 and July 13, 2021, except for the week of December 23–29, 2020 (Christmas holiday break).

### Statistical analysis

LePHIA2020 household weights were used to create LeCellPHIA base weights which were then adjusted for unequal probability of selection, non-response, and potential under-coverage of sampling frame. To create the weighted annual incidence of influenza-like illness using the LeCellPHIA data in Table 1, we created a count per exposure by sub-group by using the quasi-Poisson model (which accounted for over-dispersion), then multipled by 52 to make the outcome annual, and multipled by 100 to get annual average incidence per 100 people.

**Table 1:** Reports of influenza-like illness symptoms and estimated cumulative incidence from the LeCellPHIA participatory surveillance system, by gender, age group and region — Lesotho, July 22, 2020—July 13, 2021

	**Symptomatic Reports**^**a**^	**Total weeks reported**	**Weighted annual cumulative incidence (95% CI)**
**Men**			
Age Group			
15–19	33	6,708	30.7 (17.3 – 54.6)
20–29	119	13,852	44.1 (34.4 – 56.6)
30–39	99	11,386	45.9 (33.6 – 62.7)
40–49	77	8,239	58.2 (38.6 – 87.8)
50–59	42	4,962	46.1 (27.0 – 78.5)
60 +	125	8,926	58.5 (43.5 – 78.6)
**Total Men**	**495**	**54,073**	**46.5 (39.6 – 54.4)**
**Women**			
Age Group			
15–19	32	7,412	21.3 (13.9 – 32.7)
20–29	127	14,192	48.2 (39.3 – 59.3)
30–39	143	13,761	54.8 (43.6 – 68.8)
40–49	88	10,640	42.0 (30.4 – 58.0)
50–59	67	7,789	44.2 (30.5 – 64.1)
60 +	133	15,118	46.5 (32.9 – 65.7)
**Total Women**	**590**	**68,912**	**45.1 (39.8 – 51.1)**
**District**			
Butha Buthe	67	9,923	35.0 (21.3 – 57.6)
Leribe	185	20,115	48.4 (37.1 – 63.1)
Berea	109	13,449	40.4 (28.2 – 57.8)
Maseru	353	36,799	49.3 (41.0 – 59.4)
Mafeteng	61	11,870	26.2 (18.5 –37.2)
Mohale’s Hoek	83	8,296	55.8 (28.2 – 110.2)
Quthing	28	5,492	22.4 (11.8 – 42.2)
Qacha’s Nek	77	5,083	76.1 (56.0 – 103.4)
Mokhotlong	48	5,540	48.3 (27.9 – 83.5)
Thaba Tseka	74	6,518	56.2 (32.7 – 96.5)
**Location**			
Urban	511	54,101	49.0 (42.9 – 55.9)
Peri-Urban	110	12,465	49.5 (31.7 – 77.3)
Rural	464	56,419	42.0 (33.7 – 52.4)
**Total**	**1,085**	**122,985**	**45.7 (40.7 – 51.4)**

^a^Symptoms include fever, dry cough and shortness of breath. A report may include more than one symptom

Both the OWID and LeCellPHIA datasets were tested for normality using the Shapiro–Wilk test, and neither dataset was normally distributed. Due to the non-normal distribution of the datasets, we used a Spearman correlation to compare weekly cumulative incidence rates of LeCellPHIA ILI symptoms with the OWID count of weekly COVID-19 cases. Neither correlation test required the data to be in the same numerical format; therefore we left LeCellPHIA as a rate and the OWID data as a count.

Prior studies have used Pearson’s correlation coefficient to assess for correlation between syndromic surveillance systems and other surveillance systems [9, 22]. Therefore, in an effort to make our data comparable with previous studies, we also report the Pearson correlation coefficient between LeCellPHIA and OWID data.

Figure 2 was created by plotting weekly incidence rates of ILI symptoms reported to the LeCellPHIA surveillance system with weekly COVID-19 cases detected by the OWID surveillance system from July 22, 2020 to July 13, 2021.

### Ethics

The Lesotho National Research Ethics Committee and Columbia University Institutional Review Board (IRB) approved LeCellPHIA with exemption from committee review. The CDC IRB reviewed the protocol and deemed the research nonhuman subjects.

## Results

### Results from the LeCellPHIA syndromic surveillance system

LeCellPHIA collected 122,985 observations between July 15, 2020 – July 13, 2021. Just over half (56.0%) of the total reports were made by women (Table 1). Among men, those aged 20–29 provided the most reports (25.6%) and among women, those aged 60 and older provided the most reports (21.9%). 45.0% of reports were from people residing in a rural location, 10.1% peri-urban and 44.0% in urban locations.

Over the year, an ILI symptom was reported 1,085 times for an average annual cumulative incidence of 45.7 per 100 people (95% Confidence Interval [CI]: 40.7 – 51.4). The cumulative incidence of reports of ILI symptoms was similar among males (46.5, 95% CI: 39.6 – 54.4) and females (45.1, 95% CI: 39.8 – 51.1). Among males, the annual cumulative incidence of ILI reports was highest among persons aged 60 + (58.5, 95% CI 43.5 – 78.6). Among females, the annual cumulative incidence of ILI reports was highest among persons aged 30–39 (54.8, 95% CI: 43.6 – 68.8). There was a slightly higher annual cumulative incidence of ILI among persons living in peri-urban (49.5, 95% CI: 31.7 – 77.3) and urban compared to rural areas. Persons residing in the Qacha’s Nek district had the annual highest cumulative incidence of ILI reports (76.1, 95% CI: 56.0 – 103.4), while those residing in the Quthing district had the lowest: 22.4, 95% CI 11. 8 -42.2.

The highest incidence rate of ILI symptoms was 3.23% during the week of January 13–19, 2021 (Epi Week 3). The lowest rate (0.35%) was the week of April 21–27 (Epi Week 17) (Fig. 2).

### Results from the Our World in Data (OWID) COVID-19 surveillance system

Across the entire data collection period, 11,177 cases of COVID-19 in Lesotho were reported to the OWID surveillance system. The mean OWID weekly COVID-19 case count in Lesotho was 224; the median weekly case count for OWID was 97 (range: 1 to 2,731). The highest case count was 2,731 during the week of January 6–12, 2021 (Epi Week 2) and the lowest case count was 1 during the week of (Epi Week 13).

### Comparing weekly LeCellPHIA and OWID surveillance system

**Fig. 1:**
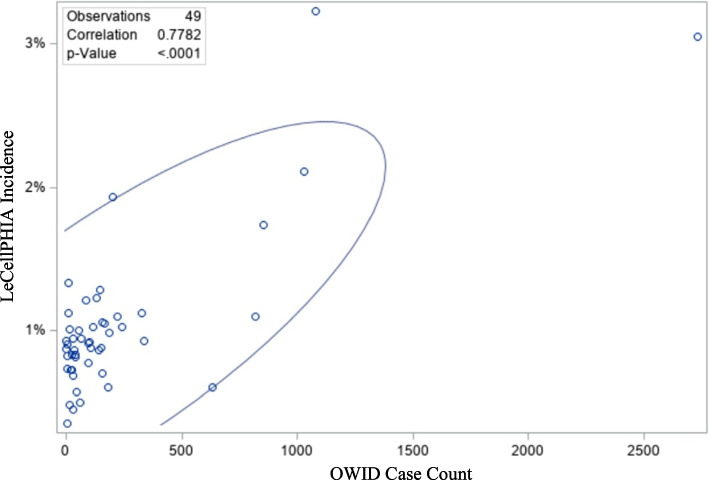
Correlation of the weekly cumulative incidence of the LeCellPHIA system with OWID case reports in Lesotho, July 22, 2020—July 13–2021

**Fig. 2:**
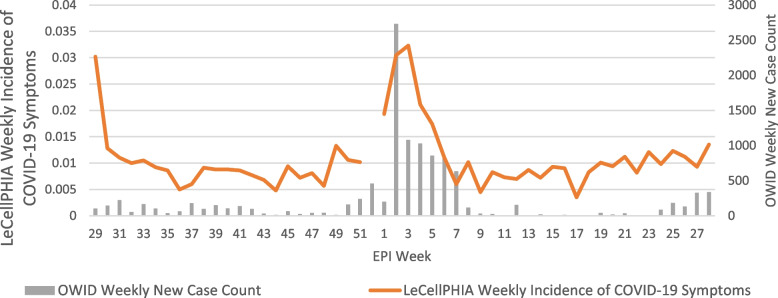
Weekly incidence rates of ILI symptoms reported to the LeCellPHIA surveillance system (orange) and weekly COVID-19 cases detected by the OWID surveillance system (grey), July 22, 2020—July 13, 2021

The Spearman’s correlation coefficient of 0.49 was statistically significant (*P* < 0.001) and indicates moderate correlation between data collected by the OWID and weekly LeCellPHIA surveillance systems [23]. Pearson’s correlation coefficient was also calculated (0.67; *P* < 0.0001), indicating statistically significant, high correlation between the OWID and LeCellPHIA datasets [23]. Figure 1 displays the correlation plot for the data from the LeCellPHIA and OWID surveillance systems across the entire data collection period. As shown in Fig. 2, the LeCellPHIA surveillance system mirrors the trends of the OWID case counts. Further, all weeks captured by the LeCellPHIA surveillance system with an ILI rate above 1.5% were between December 30, 2020 and February 02, 2021, mirroring an epidemic peak also seen in OWID data.

## Discussion

LeCellPHIA detected ILI trends consistent with OWID COVID-19 case counts and a January peak in COVID-19 case counts reported to OWID was matched by a similar peak in LeCellPHIA ILI reports. There was moderate-to-strong correlation between the two datasets. Prior studies have also found meaningful associations between syndromic surveillance systems and reference standard surveillance systems [12, 22]. More recently, prior studies have used syndromic and participatory surveillance methods to monitor the COVID-19 pandemic [24–27]. However, there are few examples from low and middle income countries; our analysis is among the first comparison of this type to focus on a low or middle income country.

The use of ILI participatory surveillance data from representatively sampled, cell phone-based systems, such as LeCellPHIA, is appealing for two reasons—the data are available in near real time, and data can be collected remotely by interviewers in a central location or in their homes. The Lesotho National COVID-19 Secretariat used LeCellPHIA national and district-level results to inform the COVID-19 Risk Adjusted strategy.

However, there are several limitations of the data that inhibit the system from detecting outbreaks and monitoring COVID-19 outbreaks. First, ILI data likely overcounts cases of COVID-19, since diseases which cause ILI symptoms, including influenza, may be included in the data. Simultaneously, given many people with COVID-19 do not exhibit symptoms, many cases are missed, and we had the main respondent report on symptoms of household members, which is less reliable than first-hand accounts. Given the lack of specificity, our approach to surveillance is best for monitoring trends rather than identifying correlates of disease. Second, the resources involved in recruiting a representative sample of a population are unavailable or infeasible in some settings. Since it has daily case counts of COVID-19 reported, OWID was the best option as a comparison dataset for LeCellPHIA but presents limitations. OWID data are not confirmed by laboratory testing; instead, these data are compiled using multiple sources including news reports and Twitter feeds and subsequently validated with local health authorities. Due to this, OWID may not be as reliable as other COVID-19 case count sources; however, OWID was the best option available.

Finally, questions related to vaccine uptake have been added to the survey since this analysis was conducted. Future analyses could be completed using these data to explore vaccine uptake and reasons for and against vaccination.

## Conclusion

Participatory surveillance is a promising complement to laboratory testing for determining disease incidence in a population, particularly since it can often be rapidly implemented with existing resources. Given that LeCellPHIA data correlated moderately-to-strongly with the reference COVID-19 data (OWID), the LeCellPHIA system may be considered as a feasible method for monitoring trends in the COVID-19 pandemic in Lesotho. Further, LeCellPHIA can be used as a model for the design of future syndromic surveillance systems which can help monitor COVID-19 outbreaks globally and potentially other disease outbreaks, as well. Finally, LeCellPHIA data are useful for implementing response to COVID-19; the Lesotho National COVID-19 Secretariat used LeCellPHIA results to inform the COVID-19 Risk Adjusted strategy.

### Supplementary Information

**Additional file 1.** Weekly Surveillance Questions (English).

## Data Availability

The datasets used and/or analysed during the current study are available from the corresponding author on reasonable request.
