# Supplementary material for: Comparison of Influenza-Like Illness (ILI) incidence data from the novel LeCellPHIA participatory surveillance system with COVID-19 case count data, Lesotho, July 2020 – July 2021
Source: BMC Infect Dis. 2023 Oct 16;23:688. doi: 10.1186/s12879-023-08664-4 (PMC10577929; doi:10.1186/s12879-023-08664-4)
Supplement: Supplementary file 1 — Additional file 1. Weekly Surveillance Questions (English). [file 12879_2023_8664_MOESM1_ESM.docx]

| **COVID-19 Symptom Questions** | | | |
| --- | --- | --- | --- |
| 8 | In the past week, have you had any flu like symptoms (e.g., fever, dry cough, shortness of breath)? | Yes 1  No 0  Hung up -6  Don’t know -8  No response -9 | If yes, go to Q8a  If no, DK or NR, go to Q9  If hung up, go to Q15 |
| 8a | Which of the following symptoms have you had? As I read one, please answer yes or no after each symptom. [Select all that apply] Have you had… | Fever? 1  Dry cough? 2  Shortness of breath? 3  Hung up -6  INAP -7  Don’t know -8  No response -9 | If any symptoms, go to 12  If no symptoms, DK or NR for all, go to 9  f hung up, go to Q15 |
| 9 | I would now like to ask about other members of your household. | (Message) |  |
| 10 | Have you seen [Household member #X] in the past week? | Yes 1  No 0  No, HH member moved permanently  No, participant moved  Hung up -6  INAP -7  Don’t know -8  No response -9 | If yes, go to 11  If no, DK or NR - re-ask Q10 until all HH members have been queried  If hung up, go to Q15 |
| 11 | In the past week, has [Household member #X] had any flu like symptoms (e.g., fever, dry cough, shortness of breath)? | Yes 1  No 0  Hung up -6  INAP -7  Don’t know -8  No response -9 | If yes, go to Q11a  If no, DK or NR, re-ask 11 until all HH members have been asked about it.  If hung up, go to Q15 |
| 11a | Which of the following symptoms has [Household member #X] had? Have they had… (read all & select all that apply) | Fever? 1  Dry cough? 2  Shortness of breath? 3  Hung up -6  INAP -7  Don’t know -8  No response -9 | If any symptoms, go to 12  If none of these symptoms, DK or NR for all, go to 13  If hung up, go to Q15 |
| 12 | Would you like me to tell you about a national toll-free hotline where you can get more information from the MoH and NACOSEC about COVID-19? It is free to call with any network. | Yes 1  No 0  Hung up -6  INAP -7  Don’t know -8  No response -9 | If yes, go to 12a  If no, go to 13  If DK or NR, go to 12d  If hung up, go to Q15 |
| 12b | The toll-free hotline phone number that is available 24 hours is 80032020, while the number that is available from 8:00am to 5:00pm is 80093030. If you get a message that the line is unavailable or a ringback tone, that means the line is busy and you should call back again. | (Message) |  |
| 13 | Do you have any questions for me about COVID-19? | Yes 1  No 0  Hung up -6  Don’t know -8  No response -9 | If yes, no, DK or NR, go to Q14  If hung up, go to Q15 |
| 14 | Thank you for sharing this information. I will call again next week. Talk to you then! | (Message) |  |
| **Questions for a respondent or HH member who had flu-like symptoms** | | | |
| 16 | You previously reported that [you/Household member #X] was ill. How is [your/Household member #X]’s health? | Same/no change 0  Better 1  Worse 2  Died 3  Recovered 4  Hung up -6  INAP -7  Don’t know -8  No response -9 | Anything but hung up, go to Q17  If hung up, go to Q15 |
| If answer to 16 = 3/died then do not include this participant in queries for future weeks.  Unless recovered/4, continue to ask #16 about HH member each week | | | |
| 17 | I would now like to ask the regular weekly questions. | (Message) | Go to Section 2 |
